# Effect of Tai Chi vs. Strength Training on Body Composition, Physical Performance, and Well-Being in Community-Dwelling Older Mexican Women

**DOI:** 10.3390/healthcare14050663

**Published:** 2026-03-05

**Authors:** Cristina Flores-Bello, Elsa Correa-Muñoz, Martha A. Sánchez-Rodríguez, Juana Rosado-Pérez, Nayeli Vaquero-Barbosa, Víctor Manuel Mendoza-Núñez

**Affiliations:** 1 Research Unit on Gerontology, FES Zaragoza, National Autonomous University of Mexico, Mexico City 09230, Mexico; rasguosaflores@yahoo.com.mx (C.F.-B.); elsa.correa@zaragoza.unam.mx (E.C.-M.); masanrod@yahoo.com.mx (M.A.S.-R.); juanarosadoperez@comunidad.unam.mx (J.R.-P.); naye7293@gmail.com (N.V.-B.); 2 Postgraduate Master’s and Doctorate in Nursing, National Autonomous University of Mexico, Mexico City 09230, Mexico

**Keywords:** Tai Chi, strength training: older adults, physical performance, body composition, cognitive functions, well-being

## Abstract

**Background/Objectives**: Tai Chi (TC) practice has been shown to positively affect the physical, psychological, and cognitive health of older adults. However, discrepancies persist regarding its effectiveness compared to strength training (ST). This study aimed to determine the effect of TC training compared to ST on body composition, physical performance, cognitive function, and psychological well-being in older adults. **Methods:** A quasi-experimental study was conducted with a convenience sample of 68 women 60 years or older, divided into three groups: (i) Tai Chi Group (TCG) n = 26; (ii) Strength Training Group (STG) n = 21; and (iii) Control Group (CG) n = 21. TCG and STG performed physical training four days a week, 60 min/day, for six months. All participants were assessed for body composition (BFP, body fat percentage; SMM, skeletal muscle mass; SMMI, skeletal muscle mass index); physical performance (4MWT, 4 m walk test; STST, sit-to-stand test; OPP, overall physical performance; HGS, handgrip strength) and Wellbeing (PWBS, psychological well-being scale of Ryff, validated for the Mexican population). The data were analyzed per protocol using repeated-measures ANOVA (TCG & STG vs. CG; TCG vs. STG), and the mean difference (MD) was calculated. **Results:** TCG showed statistically significant changes in body composition, BFP (MD, −3.4 ± 8.2, *p* < 0.05), SMM (MD, 1.6 ± 1.4, *p* < 0.001), and SMMI (MD, 0.72 ± 0.61, *p* < 0.001) after the intervention compared to CG. However, no differences were observed between TCG and STG (*p* > 0.05). Regarding physical performance, TCG showed significant changes in 4MWT (MD, −1.0 ± 1.8, *p* < 0.01) and STST (MD, −3.7 ± 4.8, *p* < 0.05) compared to CG. Differences were also observed in STST between TCG and STG (MD, −3.7 ± 4.8 vs. 0.45 ± 3, *p* < 0.05). In addition, TCG showed a significant increase in HGS (MD, 1.1 ± 1.9, *p* < 0.05) compared to CG, although no differences were observed with STG (*p* > 0.05). **Conclusions:** Our findings suggest that TC is more effective than strength training for improving body composition, physical performance, and handgrip strength in older adults living in the community.

## 1. Introduction

The World Health Organization (WHO, 2015) defines healthy aging as “the process of developing and maintaining functional ability that enables well-being in older age” [1]. This concept differs from that of health status, as its central focus is functional ability, understood as the interaction of physical, psychological, and social attributes, together with a supportive environment that allows individuals to be and do what they value in life. The WHO defines functional ability as a person’s intrinsic capacity and the characteristics of their environment. Intrinsic capacity, in turn, integrates three elements: (i) genetic inheritance; (ii) health related to aging and lifestyle; and (iii) personal characteristics. Together, these aspects reflect the sum of physical and mental capacities available to an individual in older age, which determine their ability to maintain well-being over time [1].

During the aging process, intrinsic capacity undergoes a gradual, progressive, and adaptive decline. However, when these changes reach a significant magnitude, they may become a risk factor for frailty in older age. In this context, promoting healthy lifestyles—particularly the regular practice of strength training (ST)—is relevant, as it has proven effective in preventing or delaying functional decline and reducing physical dependence and social isolation commonly associated with this condition [2]. In line with this approach, the WHO (2020) recommends engaging in 150 to 300 min of moderate-intensity aerobic physical activity per week, or 75 to 150 min of vigorous aerobic activity, as part of a comprehensive strategy to promote healthy aging [3].

In Mexico, the type of physical exercise recommended for older adults is ST [4]. In this sense, the evidence indicates that ST has positive effects on skeletal muscle mass, muscle strength, physical performance, and psychological well-being in older adults [5]. Furthermore, a systematic review and meta-analysis suggest that the benefits of physical exercise can be enhanced when combined with cognitive stimulation, as in mind–body exercise programs, where cognitive training is integrated simultaneously with bodily movements [6]. In this regard, Tai Chi (TC) stands out as a traditional Chinese exercise of moderate intensity, derived from martial arts. TC training has demonstrated benefits for physical, psychological, and cognitive health by simultaneously combining postures, breathing, and deep relaxation.

Regular TC practice is associated with a slower rate of cognitive decline, as it requires cognitive effort to perform and master complex bodily movements, and with improvements in physical performance, body composition, and vitality [7,8,9]. Its practice does not exceed 55% of maximal oxygen consumption or 60% of individual maximal heart rate, which translates into an average energy expenditure of 4 METs, that is, approximately four times the resting consumption and twice that required for slow walking. Additionally, TC improves single-leg postural control, body rotation, knee extension, and ankle plantar flexion, even with eyes closed, which is associated with up to a 47.5% reduction in fall risk. Due to its slow and low-intensity movements, TC is an ideal alternative for older adults or individuals with limitations that prevent vigorous exercise [8,9,10].

It has been reported that TC practice has a greater effect than ST on functional mobility and balance in older adults [11]; however, knowledge about the effect on body composition, specifically skeletal muscle mass and fat mass, is limited and controversial [12,13].

Our research group has conducted studies on the effects of TC training in community-dwelling Mexican older adults on oxidative stress markers and glycemic control [14,15]; however, there are no studies in our population on the effects of TC training on body composition and physical performance, or on differences relative to ST training.

In this framework, the present study aimed to determine the effect of TC training compared to ST on body composition, physical performance, and well-being in community-dwelling older Mexican women.

## 2. Materials and Methods

### 2.1. Research Design

With prior consent, a quasi-experimental study was conducted with a convenience sample, following the guidelines of the TREND statement (Transparent Reporting of Evaluations with Nonrandomized Designs) [16]. The study was approved by the Ethics Committee of the Faculty of Higher Studies Zaragoza, UNAM (Approval: FESZ/CEl/1/1/8/21/23). It was also registered on the ISRCTN platform (Registration: ISRCTN55261971) [17].

The research question in accordance with the acronym PICO (Patient, Intervention, Comparison, Outcome) was as follows: P, community-dwelling older Mexican women aged 60 to 74; I, TC training; C, ST training and group without physical exercise; O, body composition, physical performance (primary), and well-being, depressive symptoms, and cognition (secondary).

### 2.2. Setting and Population

The sample consisted of 81 older women aged 60 to 74 years, enrolled in the University Center for Healthy Aging (CUENSA, Centro Universitario para el Envejecimiento Saludable) at the Faculty of Higher Studies Zaragoza, UNAM.

Inclusion criteria: (i) no prior experience of practicing Tai Chi, (ii) no physical exercise in the last 6 months, (iii) 100% score on basic and instrumental activities of daily living instruments, (iv) independent mobility without needing assistance, (v) similar eating characteristics assessed with 24 h recall, (vi) no disabling or terminal chronic diseases, (vii) no severe cognitive impairment (Montreal Cognitive Assessment, MoCA score ≤ 23), (vii) no probable diagnosis of depression (GDS ≤ 15)

Elimination criteria: (i) people who have any musculoskeletal impairment, acute or chronic disease, (ii) individuals who did not complete the post-intervention assessments (TCG and STG) or the 6-month follow-up in the CG, (iii) individuals from the experimental groups (TCG and STG) who did not attend less than 80% of the physical exercise training.

Sample size was estimated based on the primary outcome of body composition, using data from a previous study (Hsu et al., 2015) that reported changes in lean mass and body fat following TC interventions compared with other exercise types [18]. A moderate effect size (f = 0.25), a significance level of α = 0.05, 80% statistical power, and two repeated measurements (pre- and post-intervention) were assumed. The analysis indicated an approximate requirement of 25 participants per group [18].

### 2.3. Randomization and Allocation

**Figure 1:**
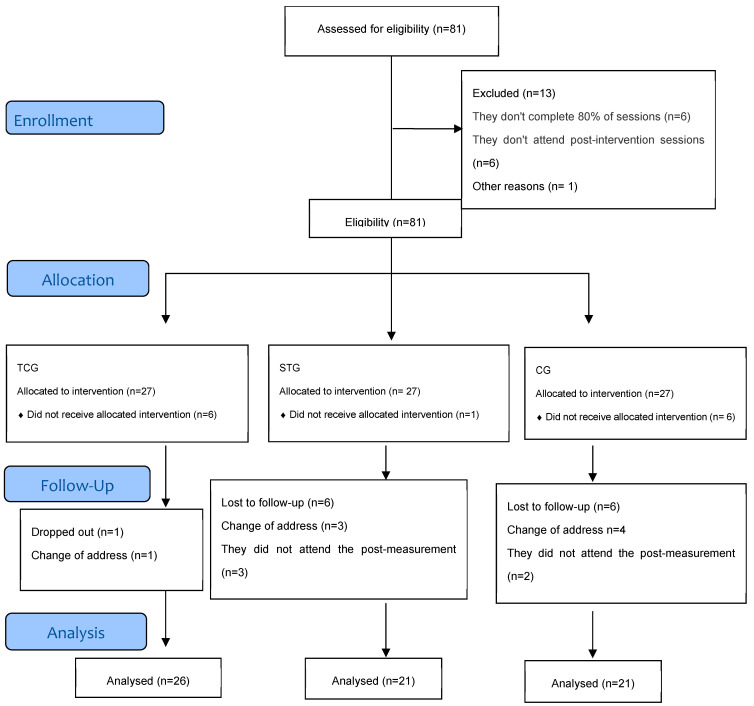
Flowchart of the participant monitoring process.

Participants who agreed to participate in the physical exercise training were randomly assigned to Tai Chi and strength training programs. At the same time, the control group consisted of individuals who, for personal reasons, could not attend the training program: (i) Tai Chi Group (TCG), n = 27; (ii) Strength Training Group (STG), n = 27; (iii) Control Group (CG), n = 27. The TCG and STG participated in a formal physical exercise training program for six months (4 days/week, 60 min per session), supervised by qualified instructors. During the intervention period, 13 participants dropped out of the study due to a change of address or failure to attend the post-measurement. Finally, 68 participants completed the study and were included in the final analysis (TCG: n = 26; STG: n = 21; CG: n = 21). Data analysis was performed according to protocol, considering the number of subjects who complied with the intervention (TCG and STG) or the follow-up time (CG) (Figure 1).

### 2.4. Measurements

All subjects in three groups were assessed before and 6 months after the intervention: blood chemistry test, physical performance, body composition, muscle strength, psychological well-being, depression, and cognitive function. All measurements before and after each group were carried out independently by different researchers.

#### 2.4.1. Blood Chemistry Test

Blood samples were obtained by venipuncture after an 8 h fast and placed in siliconized Vacutainer tubes without anticoagulant to obtain serum for blood chemistry analysis. These parameters were measured using spectrophotometric techniques on an automated clinical chemistry analyzer, Selectra Junior (Vital Scientific, Dieren, Netherlands). For all determinations, intra-assay and inter-assay coefficients of variation were below 5%. In this study, mean scores for each group are reported before and after the intervention.

#### 2.4.2. Physical Performance

Physical performance was assessed using the Short Physical Performance Battery (SPPB), which evaluates balance, gait, strength, and endurance. This test consists of three subtests: (i) Balance in three positions: feet together, semi-tandem, and tandem, each one maintained for 10 s; (ii) Lower limb strength: time required to stand up and sit down from a chair five consecutive times as quickly as possible (STST, Sit-to-Stand Test); (iii) Walking speed: time in seconds to walk 4 m at a usual pace (4MWT, 4-Meter Walk Test). It has a score of 0–12, where each domain contributes 0–4 points. Higher scores indicate higher levels of functioning [19]. This study presents the average scores and the percentage change from the baseline value after the program.

#### 2.4.3. Body Composition

To determine Body Fat Percentage (BFP), Skeletal Muscle Mass (SMM), and Skeletal Muscle Mass Index (SMMI), bioelectrical impedance analysis (BIA) was performed using a single-frequency, four-pole device (50 kHz, Quantum X, RJL System^®^, Clinton Township, MI, USA). Before assessment, participants were required to meet the following conditions: (i) fasting for at least 4 h; (ii) no alcohol consumption within 48 h before the test; (iii) no strenuous exercise within 24 h before the test. During the analysis, participants lay in a supine position while four electrodes were placed—two on the dorsum of the right hand and two on the right foot—recording resistance (R) and reactance (Xc) values [20,21].

#### 2.4.4. Handgrip Strength (HGS)

HGS was measured using a Jamar hydraulic dynamometer® (JLM JLW Instruments, Chicago, IL, USA) with a measurement range of 0–100 kg, and reliability and validity (r = 0.96) were established. The instrument was adjusted to each participant’s hand width. Three measurements were taken on each arm, with a one-minute rest interval between trials, and the maximum value obtained was recorded [22,23]. In this study, mean scores and percentage changes from baseline after the program are reported.

#### 2.4.5. Psychological Well-Being

Psychological well-being was assessed using the Ryff Psychological Well-Being Scale (PWBS), consisting of 39 items that evaluate six dimensions: (i) autonomy; (ii) personal growth; (iii) self-acceptance; (iv) purpose in life; (v) environmental mastery; and (vi) positive relations. The scale has been validated in the Mexican population with adequate overall reliability (Cronbach’s alpha = 0.918). The global score ranges from 39 to 234, with higher scores indicating greater well-being [24,25]. In this study, mean scores and percentage changes from baseline after the program are reported.

#### 2.4.6. Depression

Depression was assessed using the Geriatric Depression Scale (GDS), which consists of 30 items and has a Cronbach’s alpha of 0.94. Scores range from 0 to 30, with higher scores indicating more symptoms related to depression [26,27]. In this study, mean scores and percentage changes from baseline after the program are reported.

#### 2.4.7. Cognitive Function

Cognitive function was evaluated using the Montreal Cognitive Assessment (MoCA), a screening tool developed to detect mild cognitive impairment. It assesses visuospatial and executive abilities, naming, memory, attention, language, abstraction, and orientation, with a score range of 0–30, where higher scores indicate better cognitive performance. The scale has a Cronbach’s alpha of 0.89 for the Mexican population [28,29]. In this study, mean scores and percentage changes from baseline after the program are reported.

### 2.5. Description of the Intervention

#### 2.5.1. Physical Exercise Program

The physical activity program was conducted in groups for 6 months, with sessions lasting 1 h and held 4 times per week. It included Yang-style Tai Chi (8-form) training and muscle-strengthening exercises. The intervention was implemented at the University Center for Healthy Aging, Faculty of Higher Studies Zaragoza, UNAM. Before each session, participants’ vital signs were recorded. If any values were outside normal ranges, the participant was not allowed to engage in physical activity. Participants were also instructed to report any discomfort during or after the practice [30].

#### 2.5.2. Tai Chi Training

The intervention was delivered by personnel previously trained by an expert Tai Chi master, a member of the Mexican Academy of Chinese Martial Arts, with experience in training this population [31]. The master supervised the development of all sessions, and no adverse events related to the exercise interventions were reported during the study period (Table S1).

#### 2.5.3. Strength Training

The program was designed according to the World Health Organization (WHO) guidelines on physical activity and sedentary behavior in older adults and the International Exercise Recommendations in Older Adults—ICFSR (International Consensus) [3,32]. Instruction was provided by personnel trained by a Physical Education specialist from FES Zaragoza, UNAM, who supervised the correct execution of the training (Tables S2 and S3).

### 2.6. Statistical Analysis

Data are expressed as mean ± standard deviation. A repeated-measures analysis of variance (ANOVA) was performed (TCG & STG vs. CG; TCG vs. STG), and the mean difference (MD) was calculated. Additionally, paired-sample *t*-tests were conducted to determine within-group changes, and the percentage change from baseline was calculated. Differences were considered statistically significant at a 95% confidence level (*p* < 0.05).

To interpret the results of Eta-squared (η^2^): a partial η^2^ Eta value close to ≥0.01 is considered low, ≥0.06 medium, and a value greater than ≥0.14 large [33].

## 3. Results

### 3.1. Sociodemographic Characteristics

Table 1 shows the sociodemographic and health characteristics of the study population. Overall, the groups were similar in terms of age and sex, with only a significant difference in the percentage of participants with hypertension in the control group. However, it is important to note that all cases were under medical control.

### 3.2. Biochemical Parameters

After 6 months of intervention, most metabolic variables showed no statistically significant differences between groups. However, serum creatinine concentration decreased significantly in TCG and STG compared with the control group (*p* < 0.02) (Table 2).

### 3.3. Body Composition

TCG showed statistically significant changes in body composition, BFP (MD, −3.4 ± 8.2, *p* < 0.05), SMM (MD, 1.6 ± 1.4, *p* < 0.001), and SMMI (MD, 0.72 ± 0.61, *p* < 0.001), although no differences were observed between TCG vs. STG (*p* > 0.05) (Table 3).

### 3.4. Physical Performance and Handgrip Strength

Regarding physical performance, TCG showed statistically significant changes in 4MWT (MD, −1.0 ± 1.8, *p* < 0.01), STST (MD, −3.7 ± 4.8, *p* < 0.05), and OPP showed a non-significant trend (MD, 1.00 ± 1.6, *p* = 0.06), compared to CG. In addition, a statistically significant difference in STST was observed between the TCG vs. STG (MD, −3.7 ± 4.8 vs. 0.45 ± 3, *p* < 0.05). Likewise, TCG showed a significant increase in HGS (MD, 1.1 ± 1.9, *p* < 0.05) compared to CG, but no difference compared to STG (*p* > 0.05) (Table 4).

### 3.5. Depressive Symptoms and Cognitive Status

TCG showed a positive change in psychological well-being, with an increase in PWBS score reaching borderline statistical significance (baseline: 162 ± 27; post: 187 ± 20; *p* = 0.05). Regarding depressive symptoms, GDS scores decreased significantly in both intervention groups compared to CG: TCG (baseline: 8 ± 5; post: 7 ± 5; *p* = 0.01); STG (baseline: 10 ± 6; post: 9 ± 5; *p* = 0.001). Cognitive status, assessed by MoCA, remained unchanged in both groups (*p* > 0.5) (Table 5).

### 3.6. Percentage Change After Intervention

#### 3.6.1. Physical Performance, Muscle Strength, and Body Composition

**Figure 2:**
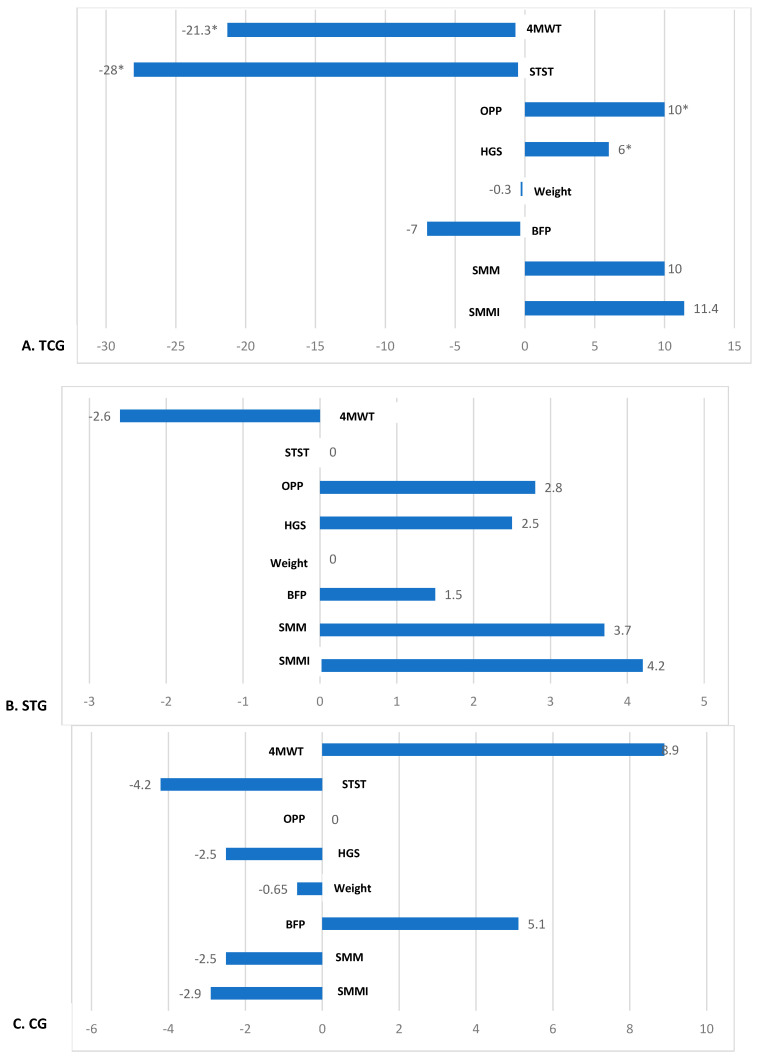
(**A**) TCG, Tai Chi Group; (**B**) STG, Strength Training Group; (**C**) CG, Control Group. Percentage changes [Δ% = (Post-pre)/pre∗100] in parameters following a six-month intervention. Related samples *t*-test, * *p* < 0.05. **4WMT**: 4 m walk test; **STST**: Sit to stand test; **OPP**: Overall physical performance; **HGS:** handgrip strength; **BFP**: body fat percentage; **SMM**: skeletal muscle mass; **SMMI**: skeletal muscle mass index.

TCG showed statistically significant percentage changes in physical performance and muscle strength tests. Specifically, reductions in time for 4MWT (−21.3%) and STST (−28%) were observed compared with STG and CG (*p* < 0.05). Likewise, this group showed an increase in OPP (+10%) and HGS (+6%) (*p* < 0.05). Modest increases in SMM (+10%) and SMMI (+11.4%) were also found, along with a reduction in BFP (−7%), although these differences were not statistically significant compared to STG and CG (*p* > 0.05) (Figure 2).

#### 3.6.2. Psychological and Cognitive Status

**Figure 3:**
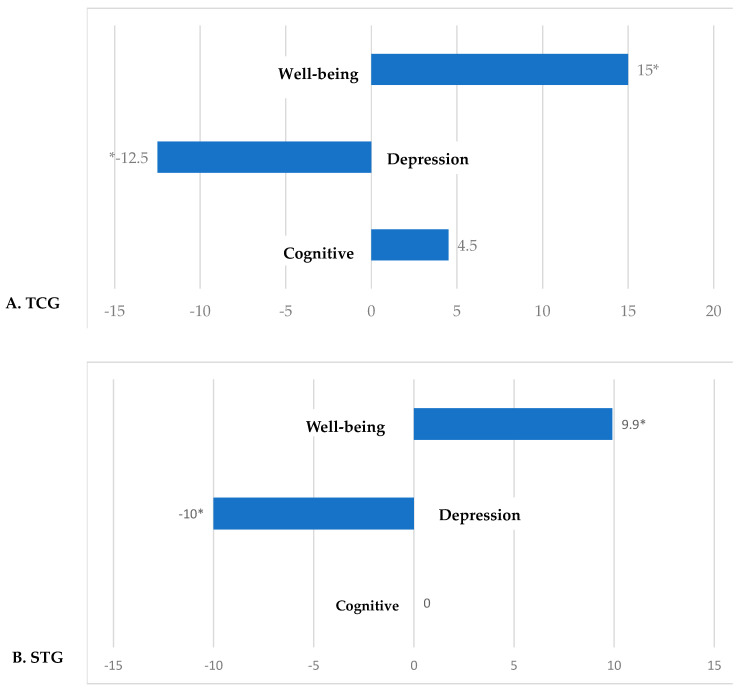

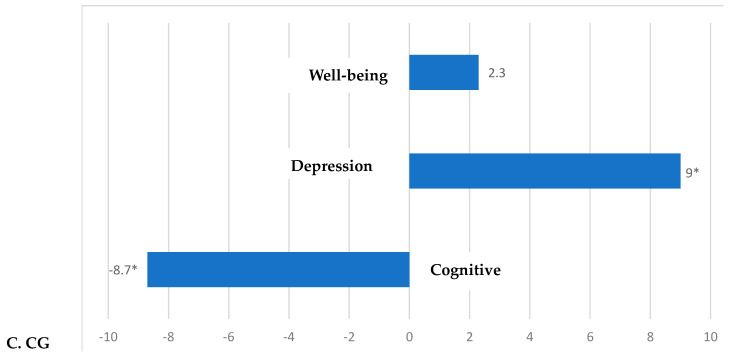
(**A**) TCG, Tai Chi Group; (**B**) STG, Strength Training Group; (**C**) CG, Control Group. Percentage changes [Δ% = (Post-pre)/pre∗100] in parameters following a six-month intervention. Related samples *t*-test, * *p* < 0.05. Wellbeing, **PWB** score: Psychological Well-Being Scales; **Depression** (GDS score, Geriatric Depression Scale); **Cognitive** (MoCA score, Montreal Cognitive Assessment).

Both TCG and STG showed a significant positive percentage change in psychological well-being, as reflected in PWBS scores (TCG: +15%; STG: +9.9%) compared to CG (+2.3%) (*p* < 0.05). Regarding depressive symptoms, both groups also showed a statistically significant reduction in GDS score (TCG: −12.5%; STG: −10%) compared to CG (*p* < 0.05). Additionally, a statistically significant increase in cognitive function was observed, as indicated by the percentage change in MoCA scores (+4.5%) compared to the control group (−8.7%) (*p* < 0.05) (Figure 3).

### 3.7. Effect Size per Group (Eta-Squared, η^2^)

Regarding the effect size, TCG showed large effects (≥0.14) on body composition parameters (SMMI, SMM, and BPF), HGS, and physical performance (OPP, STST, and 4MWT), as well as on depressive symptoms (GDS) and psychological well-being (PWB). In contrast, STG showed large effects only on depressive symptoms (GDS) and psychological well-being (PWB) (Table 6).

## 4. Discussion

Physical inactivity and muscle disuse (sedentary lifestyle) are recognized as major risk factors for the development of severe sarcopenia [34]. Furthermore, isolation and a lack of cognitive stimulation in old age are associated with a higher prevalence of cognitive decline and dementia. In this regard, social interaction related to physical exercise and the perception of goal achievement, such as self-care, positively affects psychological well-being and cognition [35]. For this reason, to interpret the effect of cognitive stimulation training as an independent intervention, we must consider the influence of social interaction, as it constitutes an intervening variable [36]. Its influence could be determined by comparing studies on cognitive stimulation training at the community level (in groups) with those on cognitive stimulation training conducted in isolation at home.

### 4.1. Body Composition

Body composition was measured through BIA with RJL System^®^, whose method is an appropriate tool for community-based screening when DXA is not feasible, enabling rapid, non-invasive, and low-cost estimation of lean and fat mass with acceptable agreement relative to DXA, thereby supporting early identification of body composition alterations associated with intrinsic capacity decline [37,38].

In our study, TCG showed a statistically significant reduction in body fat percentage and an increase in skeletal muscle mass compared to CG. Specifically, a large effect size was found for SMMI, SMM, and BPF, whereas STG showed no significant changes in body composition variables compared to CG, nor a large effect size. The positive results of TC training are similar to those previously reported in other studies [39,40]. In this regard, it has been proposed that strength training is the most suitable and effective type of physical exercise for maintaining and improving skeletal muscle mass and consequently preventing or controlling sarcopenia. This is because it involves the contraction of skeletal muscle fibers to generate work against a specific weight or external force, causing biological stress on the muscle fibers and increasing the capacity to maintain contraction (60–85% of maximum voluntary contraction) and muscle strength [41,42]. However, during this physical activity, reactive oxygen species and oxidative stress increase [43]. On the other hand, TC is a moderate physical exercise that combines deep diaphragmatic breathing with various postures, each with smooth, gentle movements. These postures require shifting body weight from one leg to the other, leading to improvements in lower body and flexor muscle strength, as well as balance. Therefore, its primary recommendation is to maintain balance and prevent falls. Furthermore, it is a physical activity that promotes mental relaxation and well-being linked to reduced oxidative stress [14,30,44,45]. In this context, we infer that the greater effect of TC training on skeletal muscle mass compared to strength training may be due to its antioxidant effects, since the loss of skeletal muscle mass during aging has been associated with increased oxidative stress [46].

Although the results suggest that TC training would be a better option to maintain or increase skeletal muscle mass in older adults living in the community, we must consider that the design of our study is quasi-experimental with some methodological limitations; therefore, the results cannot be generalized, although the findings constitute evidence to propose randomized clinical trials in representative samples.

### 4.2. Physical Performance and Handgrip Strength

In the present study, TC training for 6 months produced significant changes in physical function compared to CG. TC practitioners achieved improvements in several indicators of physical performance: (i) a significant reduction in 4MWT time; (ii) a reduction in STST time; and (iii) increases in HGS. However, only the reduction in STST time was statistically significant compared to STG. In this regard, our results are consistent with previous studies reporting the benefits of TC on mobility and physical function in sedentary older adults. For example, in a 12-week intervention, TC practitioners showed an 11.9% increase in body strength (pre: 12.4 ± 3.93; post: 13.8 ± 4.47), and reduced time in the 8-foot walk test (baseline: 7.4 s; post: 6.2 s), and improved grip strength (baseline: 25.9 kg; post: 27.9 kg) [47]. In contrast, another intervention found no significant changes in overall SPPB score, walking speed, chair rise test, grip strength, or body fat reduction after 12 weeks of TC training [48]. Similarly, a study in older adults with several comorbidities showed only trends toward improvement in SPPB after six months of TC [49]. A systematic review reported that exercise programs are effective in maintaining or improving physical performance in older adults, even in those with frailty, where the effect is more evident, likely due to a greater margin for recovery [50].

In this context, the discrepancies in results regarding the effect of TC training on physical performance and grip strength may be due to the intrinsic capabilities of different populations (genetic and epigenetic characteristics, health conditions, and sociocultural characteristics), which the WHO (2015) has identified as a determining factor in the effect of community interventions for healthy aging [1]. This underscores the importance of conducting studies with similar interventions in different contexts. However, we must also consider research designs and the measurement or control of confounding variables in these studies.

### 4.3. Psychological Status

Evidence shows that psychological well-being in older adults improves with moderate physical exercise at least twice per week, as demonstrated by changes in Ryff scale scores (baseline: 47.8 ± 6.3; post: 69.8 ± 10.8; *p* < 0.001) [51]. In this regard, TC can be classified as moderate exercise, as it does not exceed 55% of maximum oxygen intake [10]. In our study, we found that TCG had a positive effect on psychological well-being, with a 15% increase accompanied by a 12.5% reduction in depressive symptoms. These modest yet positive changes are relevant to maintaining healthy aging, given that one of the goals of community interventions is to increase healthspan associated with well-being [52]. In this regard, our findings are consistent with those of a quasi-experimental study conducted in older Korean adults, which reported a positive effect on depressive symptoms and perceived stress after eight weeks of TC training compared with a sedentary control group [53].

A positive correlation has also been observed between ST and psychological well-being, particularly in older adults [54,55]. In our study, STG showed only a significant decrease in the depressive symptom scale score compared to CG (*p* < 0.05); however, there were no changes in the psychological well-being and cognition scale scores.

Although it has been observed that TC training, by integrating movement, mindfulness, relaxation and the social component, more broadly promotes well-being and reduces depressive symptoms [56,57,58], in our study only significant changes were found in the depressive symptom scale score; however, the well-being scale score, which showed borderline statistical significance, could have significant changes with a larger sample size and/or a longer follow-up TC training period.

### 4.4. Cognitive Status

It has been reported that 36 weeks of TC training resulted in a significant increase in MoCA scores compared to the walking group (baseline: 24.67 ± 2.72; post: 23.84 ± 3.17; mean difference: 0.84 [95% CI: 0.02–1.66]; *p* = 0.046) [10]. The positive effect of TC training on cognitive function in older adults has been demonstrated in several studies [59], suggesting that TC may maintain or improve cognitive function in this population. In our study, cognitive scores remained stable when comparing the TCG and STG groups with the CG group and between the TCG and STG groups. However, in the intra-group percentage change estimate, the CG group showed a decrease in MoCA scores (−8.7%) compared to the TCG and STG groups, which maintained their scores. This behavior in cognitive function is relevant to healthy aging at the community level, since one of the objectives is to maintain functional capacity for as long as possible [1].

## 5. Limitations and Strengths

This was a quasi-experimental study in which only the type of physical exercise training could be randomly assigned; confounding variables were partially controlled for through the inclusion criteria. Furthermore, the sample is not representative, as the study was conducted in a population recruited from CUENSA (University Community Center for Healthy Aging). Hence, the people who enroll at this center are likely more aware of their health than the general older adult community. In addition, only women were included because most participants in community programs in Mexico are women, so the low participation of men in gerontology programs poses a challenge for our country and for other countries where participatory aging has been feminized, for cultural reasons. Regarding the strengths of the research, this is the first study in Mexico to show better results for TC training than ST in body composition, physical performance, and handgrip strength in older adults living in the community.

## 6. Conclusions

Our findings suggest that TC has a greater effect on body composition than strength training, particularly on skeletal muscle mass, which is linked to handgrip strength and physical performance in older adults living in the community. However, we must consider that this is a quasi-experimental study with several limitations, so the results should be confirmed with randomized clinical trials with representative samples in different contexts.

## Figures and Tables

**Table 1:** Sociodemographic and health characteristics of the study population.

Variable	TCG n = 26	STG n = 21	CG n = 21
**Age**	66 ± 6	64 ± 4	68 ± 5
**Schooling (years)**	8 ± 4	8 ± 4	9 ± 4
**Marital Status**			
In a relationship	11(42.3%)	14(66.7%)	8(38%)
Single	15(57.6%)	7(33.3%)	13(62%)
**Occupation**			
Household activities	16(61.5%)	13(62%)	14(66.7%)
Grandchild care	3(11.5%)	8(38%)	4(19%)
Community programs	7(27%)	0	3(14.3%)
**Health status**			
Healthy	16(61.5%)	16(76%)	9(43%)
DM	0	0	2(9.5%)
HBP	9(34.6%)	5(24%)	8(38%)
DM + HBP	1(3.9%)	0	2(9.5%)

**Abbreviation:** TCG, Tai Chi Group; (ii) STG, Strength Training Group; (iii) CG, Control Group (CG).

**Table 2:** Biochemical parameters by study group.

Parameters	TCG n = 26	STG n = 21	CG n = 21
**Glucose**			
Baseline	91.8 ± 9.4	99.5 ± 25	99.8 ± 21.3
Six-months	96.3 ± 8.8	102.6 ± 28	103 ± 21
Effect (MD)	7.3 ± 9.7	2.7 ± 14	−4.5 ± 15
*p*-value	0.85	0.93	
**Urea**			
Baseline	29.6 ± 7.6	28.6 ± 5.8	32.8 ± 12
Six-months	35.4 ± 9.5	37.5 ± 12	34.2 ± 12
Effect (MD)	6.1 ± 6	8.3 ± 10	1.8 ± 11
*p*-value	0.48	0.21	
**Creatinine**			
Baseline	0.95 ± 0.17	0.96 ± 0.11	0.85 ± 0.20
Six-months	0.96 ± 0.18	0.97 ± 0.15	1.04 ± 0.27
Effect (MD)	0.03 ± 0.19	0.01 ± 0.18	0.20 ± 12
*p*-value	0.002	0.005	
**Uric Acid**			
Baseline	3.7 ± 1.0	3.4 ± 0.8	3.2 ± 1.2
Six-months	3.9 ± 1.0	3.4 ± 0.7	3.7 ± 1.1
Effect (MD)	0.12 ± 0.5	−0.22 ± 0.4	0.58 ± 1.0
*p*-value	0.23	0.02	
**Cholesterol**			
Baseline	176 ± 40	183.8 ± 27	199.5 ± 31
Six-months	177 ± 37	179.5 ± 37	181.1 ± 42
Effect (MD)	0.28 ± 16	−2.3 ± 21	−19.2 ± 24
*p*-value	0.12	0.18	
**HDL**			
Baseline	61 ± 13	61 ± 11	59 ± 17
Six-months	62 ± 12	65 ± 18	63 ± 13
Effect (MD)	−1.0 ± 13	3.2 ± 7.9	0.85 ± 15
*p*-value	0.99	0.86	
**LDL**			
Baseline	67 ± 16	63 ± 18	60 ± 18
Six-months	85 ± 27	91 ± 26	96 ± 38
Effect (MD)	2.0 ± 15.8	2.7 ± 18	15.9 ± 31
*p*-value	0.26	0.28	
**Triglycerides**			
Baseline	129 ± 41	142 ± 44	134 ± 45
Six-months	113 ± 39	129 ± 49	110 ± 44
Effect (MD)	−7.0 ± 29	−12 ± 29	−20.8 ± 28
*p*-value	0.41	0.71	
**Albumin**			
Baseline	4.0 ± 0.20	4.2 ± 0.77	3.9 ± 0.22
Six-months	4.0 ± 0.24	3.9 ± 0.33	4.1 ± 0.26
Effect (MD)	0.08 ± 0.27	−0.21 ± 0.93	0.15 ± 0.27
*p*-value	0.94	0.21	

**Abbreviation:** TCG, Tai Chi Group; STG, Strength Training Group; CG, Control Group; MD, mean difference. Data are expressed as means ± standard deviation. ANOVA of repeated measures test, significance level 95%, “*p*” value (TCG & STG vs. CG). TCG vs. STG *p* > 0.05.

**Table 3:** Body composition by study group.

Parameters	TCG n = 26	STG n = 21	CG n = 21
**Weight (kg)**			
Baseline	66.5 ± 13	61.2 ± 9.0	61.5 ± 10.3
Six-months	66.7 ± 14	61.2 ± 9.4	61.1 ± 10.1
Effect (MD)	−0.07 ± 2.4	0.68 ± 2.0	−0.06 ± 1.76
*p*-value	0.97	0.64	
**BFP**			
Baseline	49.2 ± 6.1	46.2 ± 6.0	49.0 ± 4.7
Six-months	45.8 ± 6.4	46.9 ± 6.3	51.5 ± 7.0
Effect (MD)	−3.4 ± 8.2	0.74 ± 5.3	2.5 ± 9.3
*p*-value	0.045	0.76	
**SMM**			
Baseline	16.3 ± 2.6	15.9 ± 2.1	16.0 ± 3.1
Six-months	18 ± 3.0	16.5 ± 1.7	15.6 ± 3.0
Effect (MD)	1.6 ± 1.4	0.6 ± 1.8	−0.40 ± 1.6
*p*-value	<0.001	0.14	
**SMMI**			
Baseline	7.0 ± 0.8	7.1 ± 0.9	6.9 ± 1.0
Six-months	7.8 ± 0.8	7.4 ± 0.6	6.7 ± 0.9
Effect (MD)	0.72 ± 0.61	0.29 ± 0.87	0.17 ± 0.73
*p*-value	<0.001	0.13	

**Abbreviation:** TCG, Tai Chi Group; (ii) STG, Strength Training Group; (iii) CG, Control Group; BFP, Body Fat Percentage; SMM, Skeletal Muscle Mass; SMMI, Skeletal Muscle Mass Index; MD, mean difference. Data are expressed as means ± standard deviation. ANOVA of repeated measures test, significance level 95%, “*p*” value (TCG & STG vs. CG). TCG vs. STG *p* > 0.05.

**Table 4:** Physical performance and muscle strength by study group.

Parameters	TCG n = 26	STG n = 21	CG n = 21
**4MWT**			
Baseline	4.7 ± 2.2	3.9 ± 1.0	4.46 ± 1.5
Six-months	3.7 ± 0.9	3.8 ± 1.0	4.92 ± 2.5
Effect (MD)	−1.0 ± 1.8	0.005 ± 0.7	0.46 ± 1.86
*p*-value	<0.01	0.63	
**STST**			
Baseline	13.2 ± 4	11 ± 3	12 ± 6
Six-months	9.5 ± 3.2	11 ± 3	11.5 ± 6
Effect (MD)	−3.7 ± 4.8 *	0.45 ± 3	−0.3 ± 2.8
*p*-value	0.021	0.9	
**OPP**			
Baseline	10 ± 2.0	10.7 ± 1.5	9 ± 2.3
Six-months	11 ± 1.2	11.0 ± 1.2	9 ± 2.5
Effect (MD)	1.00 ± 1.6	0.33 ± 1.3	−0.05 ± 1.5
*p*-value	0.06	0.7	
**HGS**			
Baseline	19.8 ± 4	19.8 ± 4.5	20 ± 5.2
Six-months	20.9 ± 4.5	20.3 ± 4.5	19.5 ± 5.5
Effect (MD)	1.1 ± 1.9	0.7 ± 3.1	−0.85 ± 2.2
*p*-value	0.02	0.12	

**Abbreviation:** TCG, Tai Chi Group; STG, Strength Training Group; CG, Control Group; 4MWT, 4-Meter Walk Test; STST, sit-to-stand test; OPP, overall physical performance; HGS, handgrip strength; MD, mean difference. Data are expressed as means ± standard deviation. ANOVA of repeated measures test, significance level 95%, “*p*” value (TCG & STG vs. CG). * TCG vs. STG *p* < 0.05.

**Table 5:** Psychological well-being, depression, and cognitive function by study group.

Variable	TCG n = 26	STG n = 21	CG n = 21
**Wellbeing** (PWBS score)			
Baseline	162 ± 27	171 ± 23	173 ± 33
Six-months	187 ± 20	188 ± 10	177 ± 30
Effect (MD)	16.7 ± 23	7.0 ± 10	3.2 ± 13
*p*-value	0.05	0.83	
**Depression** (GDS score)			
Baseline	8 ± 5	10 ± 6	11 ± 9
Six-months	7 ± 5	9 ± 5	12 ± 9
Effect (MD)	−0.6 ± 1.2	−1.2 ± 2.1	1.0 ± 2.0
*p*-value	0.01	0.001	
**Cognitive** (MoCA score)			
Baseline	22 ± 3.9	22 ± 5.4	23 ± 4.4
Six-months	23 ± 4.9	22 ± 5.9	21 ± 4.7
Effect (MD)	0.15 ± 4.2	−0.25 ± 1.8	−1.21 ± 2.5
*p*-value	0.37	0.63	

**Abbreviation:** TCG, tai chi group; STG, strength training group; CG, control group; PWBS, psychological well-being scale; GDS, geriatric depression scale—MoCA, Montreal Cognitive Assessment; MD, mean difference. Data are expressed as means ± standard deviation. ANOVA of repeated measures test, significance level 95%, “*p*” value (TCG & STG vs. CG). TCG vs. STG *p* > 0.05.

**Table 6:** Effect size per study group.

	TCG	STG	CG
Weight	0.003	0.001	0.001
SMMI	0.59	0.10	0.05
SMM	0.56	0.10	0.06
BFP	0.34	0.02	0.07
HGS	0.26	0.05	0.13
OPP	0.28	0.06	0.01
STST	0.375	0.001	0.013
4MWT	0.238	0.001	0.062
MoCA	0.001	0.010	0.187
GDS	0.181	0.350	0.018
PWB	0.350	0.318	0.056

**Abbreviation:** TCG, Tai Chi Group; STG, Strength Training Group; CG, Control Group; SMMI, Skeletal Muscle Mass Index; SMM, Skeletal Muscle Mass; BFP, Body Fat Percentage; HGS, handgrip strength; OPP, overall physical performance; STST, sit-to-stand test; 4MWT, 4-Meter Walk Test; MoCA, Montreal cognitive assessment; GDS, geriatric depression scale); PWBS, psychological well-being scale. Eta-squared (η^2^): A partial η^2^ Eta value close to ≥0.01 is considered low, ≥0.06 medium, and a value greater than ≥0.14 large.

## Data Availability

The data presented in this study are available on request from the corresponding author. Our university states that research results information can only be provided when formally requested from the project manager.

## Supplementary Materials

The following supporting information can be downloaded at: https://www.mdpi.com/article/10.3390/healthcare14050663/s1, Table S1: Description of Tai Chi training sessions. Tables S2 and S3: Description of strength training sessions.

## Abbreviations

The following abbreviations are used in this manuscript:

TC	Tai Chi
ST	Strength training
TCG	Tai Chi group
STG	Strength training group
FP	Physical performance
CG	Control group
4MWT	4-Meter Walk Test
STST	Sit-to-Stand test
OPP	Overall physical performance
MS	Muscle strength
W	Weight
BFP	Body Fat Percentage
SMM	Skeletal Muscle Mass
SMMI	Skeletal Muscle Mass Index
PWBS	Psychological Well-Being Scale
GDS	Geriatric Depression Scale
MoCA	Montreal Cognitive Assessment
WHO	World Health Organization
Mets	Metabolic equivalents
