# Supplementary material for: Effect of Tai Chi vs. Strength Training on Body Composition, Physical Performance, and Well-Being in Community-Dwelling Older Mexican Women
Source: Healthcare (Basel). 2026 Mar 5;14(5):663. doi: 10.3390/healthcare14050663 (PMC12985254; doi:10.3390/healthcare14050663)
Supplement: Supplementary file 1 [file healthcare-14-00663-s001.zip › healthcare-4094897-supplementary.pdf]

Table S1. Supplementary Materials S1. Description of Tai Chi training sessions

<table>
<tr><th>Stages of each session</th><th>Yang-style Tai Chi 8 form</th></tr>
<tr><td>Warm-up (10 minutes): Joint-lubricating stretching movements.

Main exercise (40 minutes): Movements performed in standing position with knee flexion.

Cool-down (10 minutes): Static stretching and controlled breathing exercises to return the body to a state of physiological relaxation.</td><td>Opening: Prepares body and mind to begin practice.

• Posture 1: Repulse the Monkey: backward movement alternating arms, one pushing forward while the other retracts.
• Posture 2: Brush Knee: the body shifts forward, one arm brushes the knee while stepping forward with trunk rotation and weight transfer.
• Posture 3: Parting the Wild Horse's Mane: lateral stepping; the foot advances and one arm remains at shoulder height while the other is placed lower at waist height.
• Posture 4: Cloud Hands: continuous, wave-like arm movement accompanied by side-to-side weight shifting.
• Posture 5: Golden Rooster Stands on One Leg: body supported on one leg while lifting the opposite leg.
• Posture 6: Heel Kick: leg extension with a front kick.
• Posture 7: Grasp the Sparrow's Tail: begin with rear leg, move the waist and front hand.
• Posture 8: Cross Hands: hands join in front of the body and lower toward the sides, achieving a calm state and signaling the end of the routine.

Closing: Return to initial posture, stabilize breathing and body at the end of practice.

Each movement was repeated between 8 and 10 times at a slow, controlled rhythm. Once participants mastered the basic postures, both static and dynamic, they began linking the eight Tai Chi postures into a continuous sequence. Each daily session included at least five full repetitions of the Tai Chi sequence, in addition to individual practice of each movement</td></tr>
</table>

Table S2. Supplementary Materials S2. Description of strength training sessions

Stages of each session	Strength training (main exercise)
**Warm-up (10 minutes):** included 5 minutes of dynamic stretching to improve joint mobility and 5 minutes of cardiovascular activation to gradually increase heart rate and prepare the neuromuscular system.<br><br>**Main exercise (40 minutes):** focused on developing muscular strength, power, and cardiorespiratory endurance using global exercises involving major muscle groups of both upper and lower body related to physical function and mobility.<br><br>**Cool-down (10 minutes):** consisted of static stretching and controlled breathing exercises to facilitate muscle recovery and promote a gradual return to a physiologically relaxed state.	**Weeks 1 to 12:** The program focused on seated exercises and bodyweight movements, emphasizing adaptation, technical skill acquisition, and initial progression.<br><br>**Weeks 13 to 24:** Standing exercises, wall-based bodyweight movements, and light external resistance (dumbbells, low-intensity elastic bands, and balls) were introduced. The exercise framework remained consistent, while execution quality improved, with increased tolerance to effort and individualized adjustments in pace, resistance, and continuity.<br><br>**Volume and intensity** were progressively increased through additional blocks, sets, and exercise complexity, while respecting each participant's individual tolerance.

Table S3. Supplementary Materials S3. Description of strength training sessions

Weeks	Main Objective	Type of exercises in main session (40 min)	Organization / Progression
1–2	Neuromuscular adaptation and functional mobility	Exercises in chair and standing with body weight: assisted squat, leg extensions, heel raises, trunk mobility	1 block, 2 to 3 series, 10 to 12 repetitions, 30 seconds, emphasis on technique and familiarization
3–4	Beginning of functional strength and coordination	Exercises with chair and on wall: wall push-ups, assisted static squat, leg swings, trunk twists	2 blocks, 3 series, 12 to 15 repetitions, 30 to 40 seconds, increase in volume, structured breaks
5–6	Increase in strength and muscular endurance	Introduction of light external resistance (dumbbells 0.5 to 1 kg): shoulder press, functional squat, boxing punches, standing abdominals	2 to 3 blocks, 3 series, 15 repetitions, 40 seconds, greater complexity and postural control
7–8	Combined work strength–endurance–balance	Standing and mat exercises: functional circuits, dynamic balance, core and lower body work.<br><br>**a) Functional circuit:**<br>Sit-to-Stand from a chair (sit down–stand up)	3 blocks, 3 series, 15 repetitions, 40 seconds, greater continuity of effort.

		-Marching in place raising knees<br>- Side step with chair support<br>-Step up and down from a low step<br>**b) Balance with chair or wall support**<br>-Leg swing forward–backward<br>-Walking in a straight line (heel–toe)<br>- Step forward and backward with weight shift<br>**-** Reaches with arm maintaining single-leg support<br>-Controlled 180° turn around a cone<br>**C) Core work on mat:**<br>Glute bridge<br>-Isometric abdominal (transverse activation)<br>-Alternate leg raises with bent knees<br>-Modified plank (knees supported)<br>-Pelvic tilts in supine position<br>**d) Lower body:**<br>-Partial squat<br>-Heel and toe raises<br>-Hip extension in standing position<br>-Hip abduction with support<br>-Straight leg raise on ma	
9–10	Consolidation of global endurance and strength	Multicomponent circuits with light external resistance (dumbbells, elastic band, balls, wall), coordinated arm and leg movements, dynamic work<br><br>**a) With dumbbells**<br>-Shoulder Press<br>-Squat with Bicep Curl<br>**-** Dynamic marching with lateral raise<br>-Boxing punch<br>-Sit-to-Stand with load<br>**b) With Elastic Bands:**<br>-Side step with arm opening<br>-Row with elastic band<br>-Row plus side step<br>-Squat with band on knees and front push<br>--Marching with elastic band	3 blocks, 3 series, 15 to 20 repetitions, 45 seconds, 1 to 2 minutes rest, increase in effective working time

		**c) With Balls:**<br>-Squat with wall support<br>-Knee raise with ball touch<br>-Sit-to-Stand<br>**d) Push to the wall:**<br>-Wall push-up both arms<br>-Push-up with right arm on wall<br>-Push-up with left arm on wall<br>-Heel raise at the same time<br>**e) Dynamic work**<br>-Continuous marching with changes of direction<br>-Step forward–backward with trunk rotation<br>-Alternate knee raises with active arms<br>-Walking with light load in hands<br>-Tandem walking with trunk rotation<br>-Step over obstacle<br>-Cross March (elbow-knee)<br>-Heel raise with arm opening	
11–12	Functional integration and maintenance	Integrated full-body circuits, emphasis on functional autonomy and movement control:	3 blocks, 3 series, 15 to 20 repetitions, 45 seconds, 1 to 2 minutes rest, moderate intensity, individual adjustment
13–24	Program repetition (2nd cycle)	Repetition of the weeks 1–12 scheme with individual progression (greater control, better tolerance to effort)	3 to 4 blocks, 3 series, 15 to 20 repetitions, 45 seconds, 1 to 2 minutes rest, adjustment of loads, rhythm and intensity according to tolerance
